# Supplementary material for: Using single cell atlas data to reconstruct regulatory networks
Source: Nucleic Acids Res. 2023 Feb 10;51(7):e38. doi: 10.1093/nar/gkad053 (PMC10123116; doi:10.1093/nar/gkad053)
Supplement: gkad053_Supplemental_Files [file gkad053_supplemental_files.zip › supp_file1_rev1.pdf]

Supplementary Information

Using single cell atlas data to reconstruct regulatory networks

Qi Song[1], Matthew Raffalo[1], Ziv Bar-Joseph[12*]

[1]Computational Biology Department, School of Computer Science, Carnegie Mellon University, Pittsburgh, PA 15213, USA

[2]Machine Learning Department, School of Computer Science, Carnegie Mellon University, Pittsburgh, PA 15213, USA

* To whom correspondence should be addressed.

Qi Song: qisong@andrew.cmu.edu

Ziv Bar-Joseph: zivbj@cs.cmu.edu

MTLRank constructs cell-type-specific networks

One unique advantage of scRNA-seq technology is that it enables the investigation of molecular activities in heterogenous cell populations. While the focus of HuBMAP consortium is generating tissue-level molecular maps, the advantage of our pipeline is that it enables the construction of cell-type-specific networks from the tissue level model. This could be achieved by summing up the absolute values of SHAP scores for any sub-population of cells (based on their cell types) and construct GRNs based on the aggregated scores. To demonstrate cell-type level analysis using our framework, we present here an application of this approach to a HubMap Slide-seq left kidney data set using cell type labels provided by Azimuth (1) (https://portal.hubmapconsortium.org/browse/dataset/29596bee3f70b2bfc89d184fe38a3daa). We used the same scATAC-seq / ChIP-seq data as we used in the previous analysis. While cell-type-specific networks are not known for many cell types, we used known kidney TFs to analyze the results. Specifically, we focused on the Kruppel like factor (KLFs) family genes, which are known to regulate kidney disease (2). Cell-type-specific GRNs were constructed using the same procedure in the previous analysis but only within each cell type population. Next, we investigated the degrees of KLF genes in the GRNs of various kidney cell types. We found that KLF4 is predicted to actively regulate many other genes in different cell types, particularly in endothelial, fibroblast, and collecting duct cells (Fig. S4). This observation is consistent with previous reports that KLF4 is actively involved in anti-inflammatory response in endothelial cells (3) and in development of renal fibrosis (3). The other notable KLF family gene is KLF2. We found high network degrees of KLF2 in neutrophil, granular, and endothelial cells, suggesting its important roles in these cell types (Fig. S4). Previous findings have confirmed that KLF2 is a regulator for neutrophil activation (4), and a regulator for endothelial barrier function (5). These results show that our framework can successfully recover important cell-type-specific regulators from a given subset of inputs.

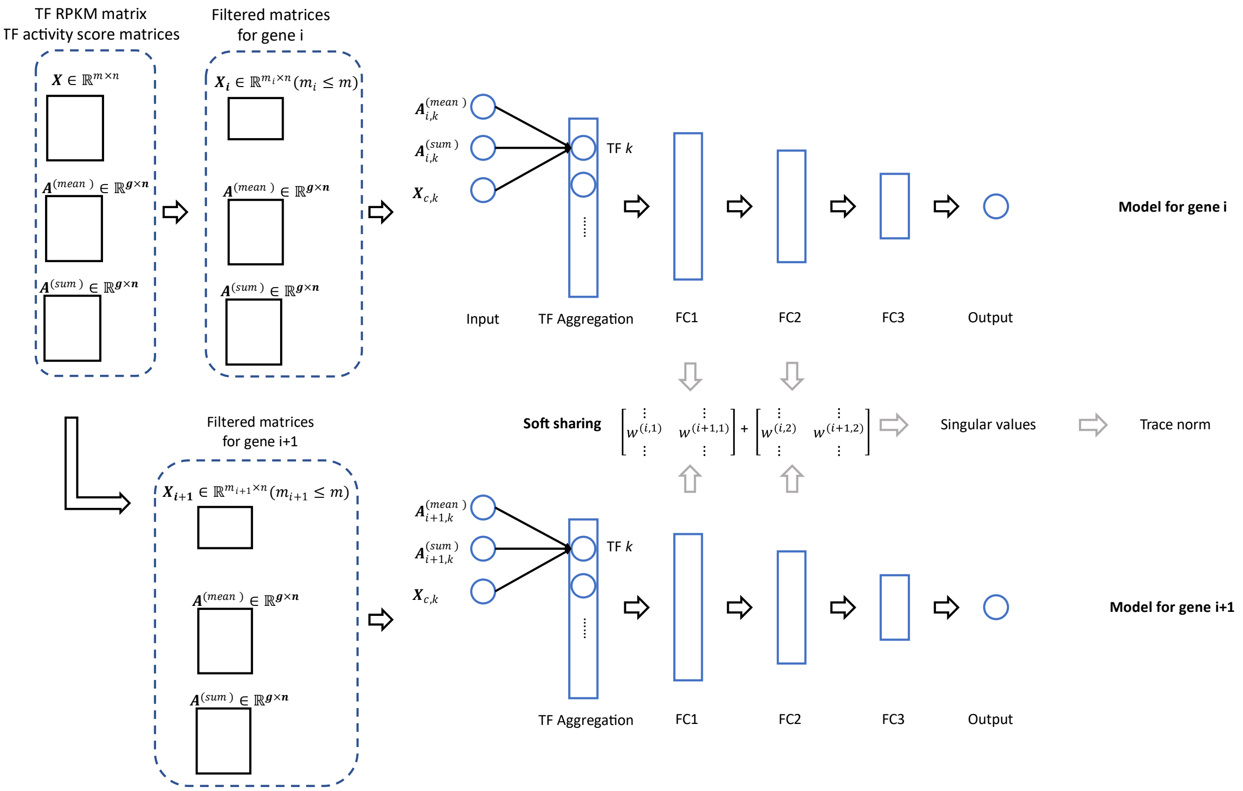

Figure S1. Training scheme and model architecture of MultiRank. This figure shows an example of training two genes using MultiRank framework. The three rectangles with blue dash line borders represent input training data. Training data was specifically filtered for each target gene to predict its velocities. $\boldsymbol{X}$ is the original RPKM matrix with m cells and n TFs. After gene-specific filtering, each target gene i is assigned with a sub RPKM matrix $\boldsymbol{X_i}$ with m_i cells and n TFs. m_i is the number of cells with available velocities for target gene i. Activity score matrices $\boldsymbol{A}^{(sum)}$ and $\boldsymbol{A}^{(mean)}$ has the same shape $g \times n$ for all target genes, where g is equal to the number of all target genes. The input layer of the model aggregates the activity scores and TF RPKM expressions for each TF. Then input layer is followed three fully connected layers FC1, FC2, FC3, and an output layer that predicts the velocities of target gene i. Trace norm regularization-based parameter soft sharing was performed on FC1 and FC2 across the two tasks. In our study, the same parameter sharing strategy was applied to gene clusters with more than two genes.

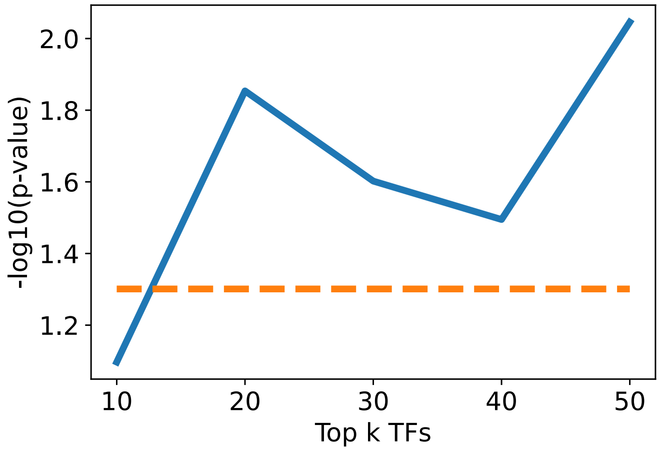

Figure S2. Negative log10 p-value for the enrichment of c-Jun related TFs among the top k selected TFs by MTLRank. GRN (gene regulatory network) was constructed from a TF perturbation dataset where c-Jun was perturbed to induce expression response in human CAR-T cells. We used scRNA-seq and ATAC-seq data generated from the CAR-T cells, and the ChIP-seq data collected as described in the main manuscript as inputs to train the models. GRN was constructed following the same protocol as described in the main manuscript. Blue line indicates the negative log10 p-value for the significance level of enrichment of c-Jun related TFs and yellow line indicates the negative log10 p-value where p-value is equal to 0.05.

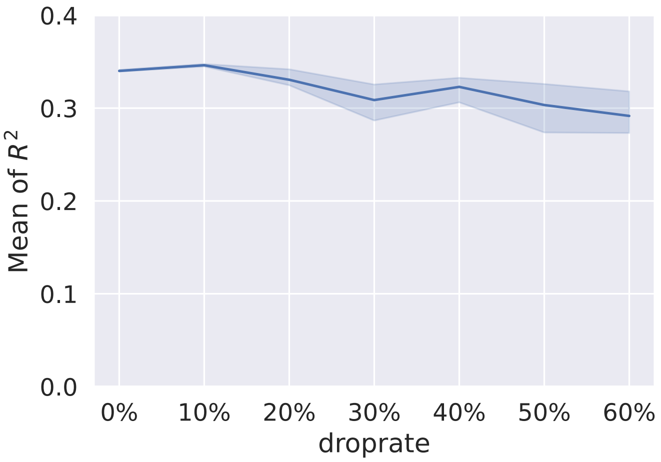

Figure S3. The mean of R^2 scores under different drop-out rate. We performed the random drop-out experiment in liver data. Under each drop-out rate, we randomly set expressions of genes in the given percentage of cells to zero and repeated this procedure by three times. This means the average drop-out rate for all genes is at least bigger than the given percentages on x-axis. The blue

line indicates the mean of R^2 among all tested genes and the light blue area around the line indicates the standard deviations among the three replicates.

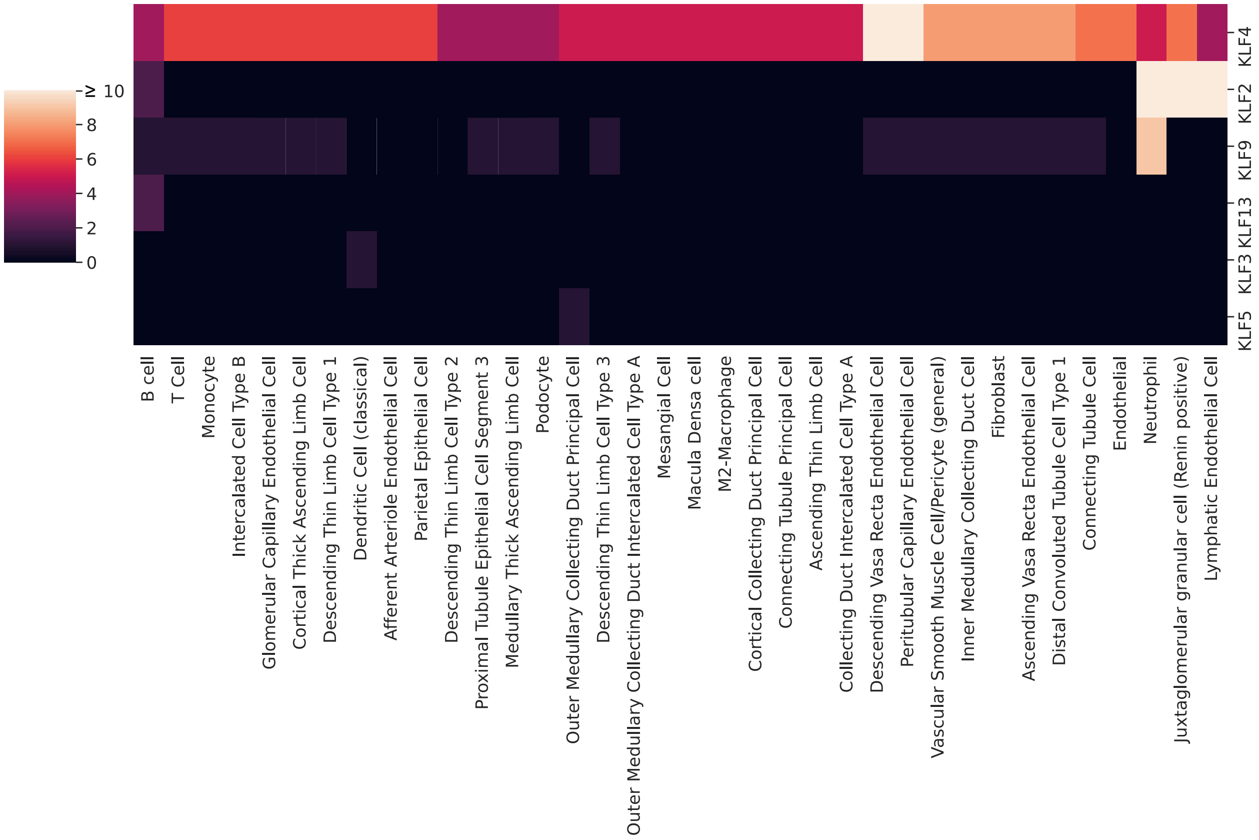

Figure S4. The cell-type-specificity activities of KLF family genes. We constructed cell-type-specific GRNs for an annotated left kidney scRNA-seq data set. We trained MTLRank models using all single cells in this data set and aggregated the absolute values of SHAP scores separately for each cell type to construct cell-type-specific GRNs. Colors scale in the plot indicates the degree of each KLF gene in the corresponding cell-type-specific GRN. It is observed that KLF4 and KLF2 showed distinctive cell-type-specific activities.

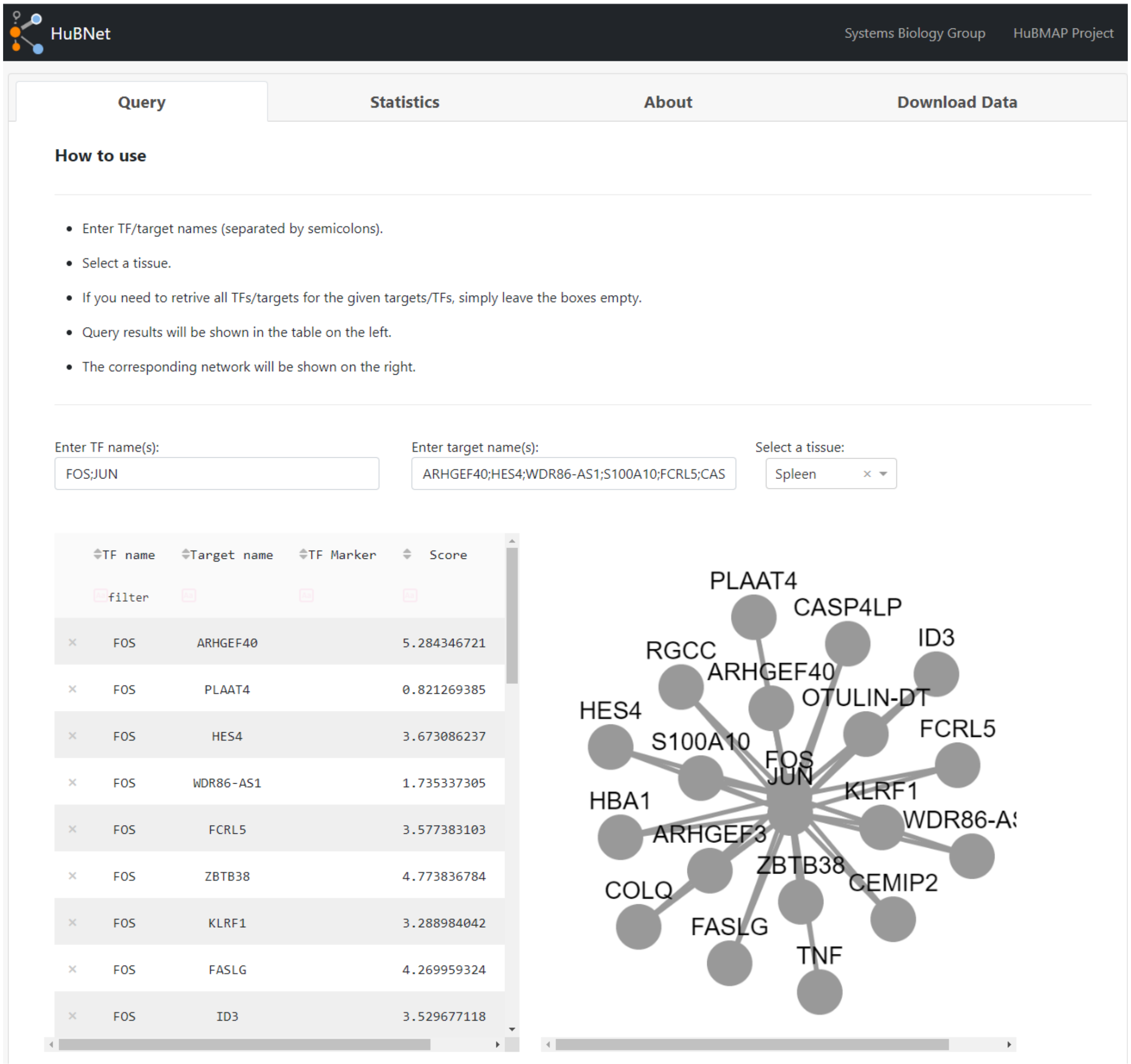

Figure S5. In user interface of the web portal HuBNet. User may query the interactions among given TFs and genes in a given tissue. Interactions will be shown in the table below and visualization of the interactions will be shown right next to the table.

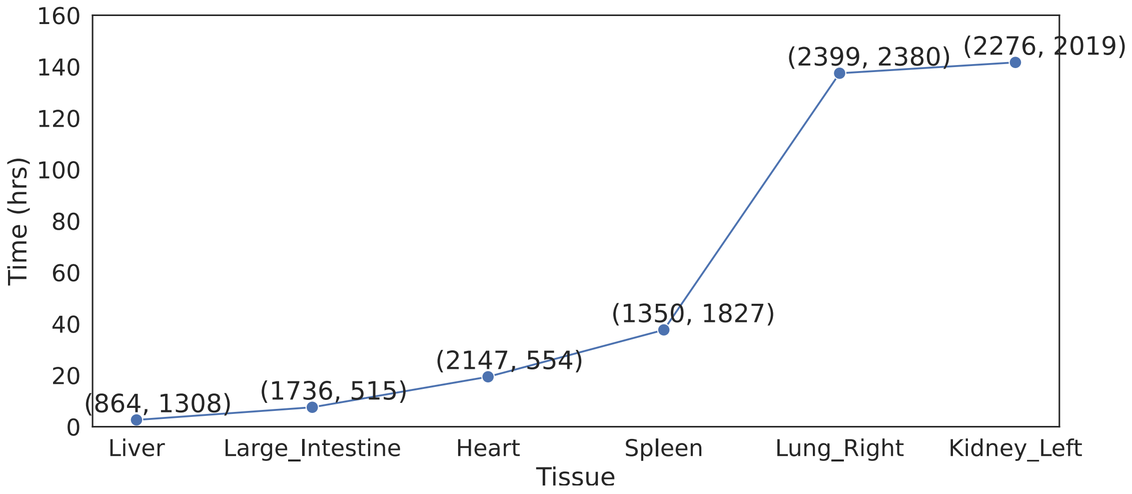

Figure S6. Running time for each tissue. For each time, running time is considered as the sum of running time for model training and TF ranking based on SHAP computation. Numbers listed in parentheses indicate total number of TFs and target genes used in that tissue.

Table S1. Meta data of donors.

HuBMAP ID	Data type	Race	Age	Gender	Tissue
HBM492.JRZX.537	scATAC-seq	White	51	Male	Liver
HBM492.JRZX.537	scATAC-seq	White	55	Female	Liver
HBM889.RSJQ.447	scATAC-seq	White	25	Female	Heart
HBM492.JRZX.537	scATAC-seq	White	55	Female	Heart
HBM896.VZXP.254	scATAC-seq	White	43	Male	Heart
HBM985.MBDB.567	scATAC-seq	Black/African American	60	Male	Heart
HBM427.KCLS.937	scATAC-seq	White	53	Male	Heart
HBM724.WJQQ.227	scATAC-seq	White	51	Male	Heart
HBM349.DLJD.259	scATAC-seq	White	43	Female	Heart
HBM826.RSPW.849	scATAC-seq	White	49	Female	Heart
HBM546.MHVZ.749	scATAC-seq	White	50	Male	Heart

HBM694.QDFG.746	scATAC-seq	Black/African American	78	Male	Large intestine
HBM765.TRHD.452	scATAC-seq	White	24	Female	Large intestine
HBM985.MBDB.567	scATAC-seq	Black/African American	60	Male	Spleen
HBM985.MBDB.567	scATAC-seq	Black/African American	60	Male	Right lung
HBM697.XFCZ.924	scRNA-seq	Unknown	44	Male	Liver
HBM527.VDJP.733	scRNA-seq	Black/African American	26	Male	Liver
HBM796.MXZR.526	scRNA-seq	White	65	Male	Liver
HBM369.SGNG.277	scRNA-seq	White	41	Female	Liver
HBM889.RSJQ.447	scRNA-seq	White	25	Female	Heart
HBM796.ZVVX.764	scRNA-seq	White	45	Male	Heart
HBM984.MHHN.738	scRNA-seq	White	66	Male	Left kidney
HBM773.MKPP.732	scRNA-seq	White	69	Female	Left kidney
HBM485.KRGM.628	scRNA-seq	White	76	Male	Left kidney
HBM277.DBXJ.256	scRNA-seq	White	25	Female	Left kidney
HBM277.XCLT.589	scRNA-seq	White	63	Female	Left kidney
HBM277.DBXJ.256	scRNA-seq	White	25	Female	Left kidney
HBM295.GHMR.582	scRNA-seq	White	54	Male	Left kidney
HBM796.VGTX.823	scRNA-seq	White	14	Female	Left kidney

HBM584.TVKC.257	scRNA-seq	White	45	Female	Left kidney
HBM765.TRHD.452	scRNA-seq	White	24	Female	Large intestine
HBM694.QDFG.746	scRNA-seq	Black/African American	78	Male	Large intestine
HBM966.VNKN.965	scRNA-seq	White	20	Male	Spleen
HBM298.KGNJ.374	scRNA-seq	Black/African American	21	Female	Spleen
HBM245.ZWNT.288	scRNA-seq	Hispanic	10	Male	Spleen
HBM549.RJKF.748	scRNA-seq	White	18	Male	Spleen
HBM966.VNKN.965	scRNA-seq	White	20	Male	Spleen
HBM298.KGNJ.374	scRNA-seq	White	21	Female	Spleen
HBM943.SCQQ.877	scRNA-seq	Black/African American	37	Male	Right lung
HBM584.TVKC.257	SNARE-seq	White	45	Female	Left kidney
HBM289.BLHF.363	SNARE-seq	White	57	Female	Right kidney
HBM943.SCQQ.877	SNARE-seq	Black/African American	37	Male	Right lung

Table S2. Hyperparameters for MultiRank.

Parameter	Value	Note
α	0.01	learning rate
γ	0.01	trace norm strength
λ	0.01	L1 regularization strength
batch size	256	NA
activation function	ReLU for layer TF aggregation, FC1,FC2, and FC3; linear for output layer	NA

epochs	8 for liver<br>4 for other tissues	NA

* Larger value of γ might be set. However, this could lead to loss of specificity for each target gene.

Table S3. Hyperparameters for other methods.

Method	Parameter	Value	Note
LASSO	α	0.1	L1 regularization strength
GENIE3	number of estimators	1000	NA
	regression model	random forest	NA
	number of features for splitting node	square root of the number of input TFs	NA
GRNBoost2	learning rate	0.01	NA
	number of estimators	5000	NA
	number of features for splitting node	10% of number of input TFs	NA
	the fraction of samples to be used for fitting each estimator	90%	NA
CellOracle	α	1	regularization strength

Table S4. Number of ground truth TFs in each tissue.

	Liver	Heart	Left kidney	Large intestine	Spleen	Right lung
Ground truth TFs	48	33	59	17	0*	95

* Zero indicates that TF-Marker database does not contain any tissue-specific TFs for spleen tissue

Table S5. Overrepresented GO terms for FOS-JUN target genes.

GO	FDR(BH)	Study count	Population count	Enrich fold
negative regulation of interleukin-2 production	0.001	7	26	14.93642072
negative regulation of mitotic cell cycle phase transition	0.001	4	4	55.47813411
T cell activation	0.005	8	49	9.057654549
T cell differentiation	0.005	7	36	10.78741497
negative regulation of T cell mediated cytotoxicity	0.01	4	8	27.73906706

References

1. Hao,Y., Hao,S., Andersen-Nissen,E., Mauck,W.M., Zheng,S., Butler,A., Lee,M.J., Wilk,A.J., Darby,C., Zager,M., *et al.* (2021) Integrated analysis of multimodal single-cell data. *Cell*, 10.1016/j.cell.2021.04.048.

2. Rane,M.J., Zhao,Y. and Cai,L. (2019) Krüppel-like factors (KLFs) in renal physiology and disease. *EBioMedicine*, 10.1016/j.ebiom.2019.01.021.

3. Hamik,A., Lin,Z., Kumar,A., Balcells,M., Sinha,S., Katz,J., Feinberg,M.W., Gerszten,R.E., Edelman,E.R.

and Jain,M.K. (2007) Kruppel-like factor 4 regulates endothelial inflammation. *J. Biol. Chem.*, 10.1074/jbc.M700078200.

4. Tang,X., Wang,P., Zhang,R., Watanabe,I., Chang,E., Vinayachandran,V., Nayak,L., Lapping,S., Liao,S., Madera,A., *et al.* (2022) KLF2 regulates neutrophil activation and thrombosis in cardiac hypertrophy and heart failure progression. *J. Clin. Invest.*, 10.1172/JCI147191.

5. Lin,Z. (2010) KLF2 regulates endothelial barrier function. *Arterioscler. Thromb. Vasc. Biol.*
